# Acute retinal necrosis following dexamethasone intravitreal implant (Ozurdex®) administration in an immunocompetent adult with a history of HSV encephalitis: a case report

**DOI:** 10.1186/s12886-020-01514-w

**Published:** 2020-06-22

**Authors:** Zhi-Yong Zhang, Xiu-Yun Liu, Tao Jiang

**Affiliations:** 1 Eye Center, Second Affiliated Hospital, School of Medicine, Zhejiang University, Hangzhou, 310009 Zhejiang People’s Republic of China; 2 Zhejiang Chinese Medical University, Hangzhou, Zhejiang People’s Republic of China

**Keywords:** Acute retinal necrosis, Intravitreal injections, Dexamethasone intravitreal implant, Retinal vein occlusion

## Abstract

**Background:**

Dexamethasone intravitreal implants (0.7 mg) (Ozurdex®, Allergan Inc., Madison, NJ) are FDA approved for managing macular oedema (ME) of retinal vein occlusion (RVO). The major complications associated with intravitreal Ozurdex® implant include increased intraocular pressure and cataract progression. In regard to the occurrence of retinal complications, we report an unusual intravitreal Ozurdex® implantation-related acute retinal necrosis (ARN).

**Case presentation:**

A 45-year-old immunocompetent woman with a history of encephalitis presented with photophobia, redness, floaters, and rapidly decreased vision in her left eye. Three and six months ago, she received two doses of intravitreal Ozurdex® implant for ME of RVO. Clinical evaluation, including slit-lamp biomicroscopy, retinal photography, and fluorescein angiography, revealed anterior chamber cells, granulomatous keratic precipitates, cells in the vitreous, optic disc oedema, occlusive retinal vasculitis, scattered retinal haemorrhages, one quadrant of peripheral white areas with retinal necrosis, optic disc and vessels fluorescein staining, and retinal nonperfusion zones. All the above clinical manifestations showed an ARN. Herpes simplex virus was detected in the aqueous and vitreous humour by quantitative polymerase chain reaction testing. Intravenous acyclovir 500 mg tid for 7 days followed by oral valcyclovir was immediately performed for ARN. At 4 months, the patient’s condition improved without retinal detachment, and the best-corrected visual acuity remained stable at 0.3.

**Conclusions:**

ARN might represent a risk of Ozurdex® administration.

## Background

Retinal vein occlusion (RVO) is the second-most common retinal vascular disease after diabetic retinopathy [1, 2]. Macular oedema (ME) is the most common cause of reduce vision after RVO [2, 3]. Dexamethasone intravitreal implant (Ozurdex®) injections are effective in managing RVO-related macular oedema by reducing vascular permeability and inhibiting the secretion of inflammatory mediators [4, 5]. The complications associated with intravitreal Ozurdex® injections in previous reports have mainly included increased intraocular pressure and cataract progression [6–8].

ARN is an uncommon necrotizing retinitis, with clinical characteristics presenting as focal and well-demarcated areas of retinal necrosis located in the peripheral retina, the rapid and circumferential progression of necrosis, evidence of occlusive vasculopathy, and a prominent inflammatory reaction in the vitreous and anterior chamber [9]. Etiologic confirmation via intraocular fluid analysis has shown that varicella zoster virus (VZV), herpes simplex virus (HSV) type-1 and -2, cytomegalovirus (CMV), and Epstein-Barr virus (EBV) are related to the pathogenesis of ARN [10, 11]. It is widely known that iatrogenesis immunosuppression associated with subtenonial or intravitreal corticosteroids may predispose patients to ARN, and several reports of ARN have shown its relation to intravitreal Ozurdex® injection [12–14]. Here, we report a case of intravitreal Ozurdex® injection-related ARN in an immunocompetent patient with RVO.

## Case presentation

**Fig. 1:**
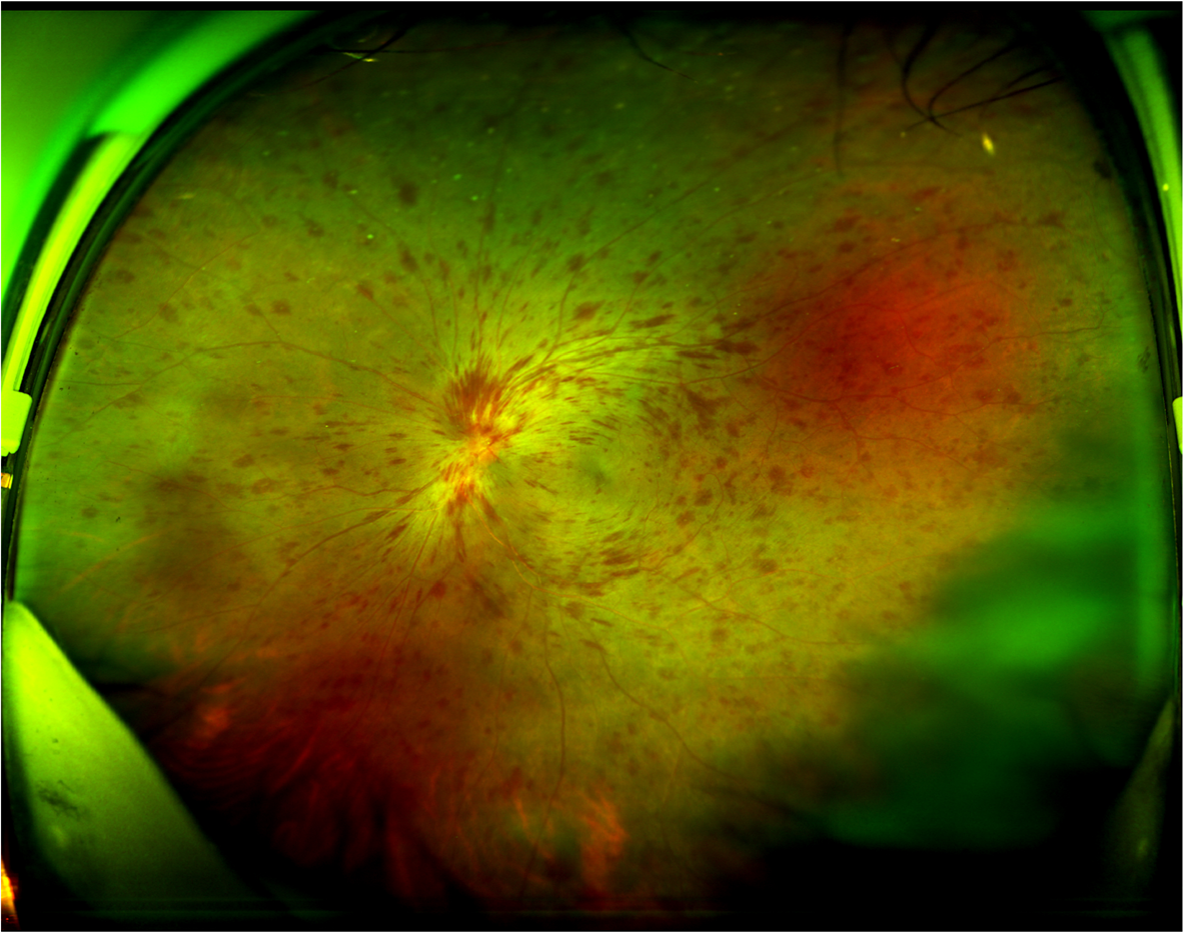
Ultra-widefield fundus photography of the left eye at initial presentation showing numerous retinal haemorrhages, vascular dilation and tortuosity, and optic disc oedema consistent with central retinal vein occlusion

**Fig. 2:**
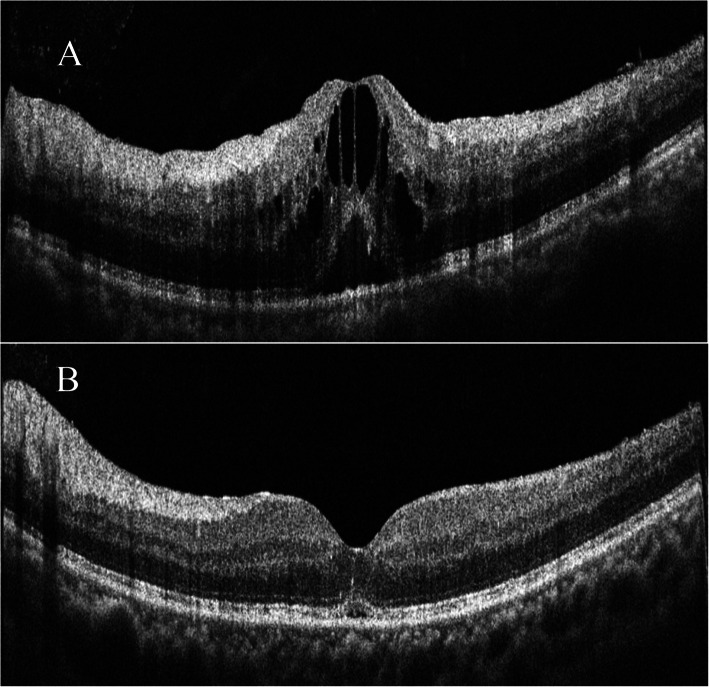
Optical coherence tomography (OCT) of left eye showing (**a**) cystoid macular oedema at initial presentation with a diagnosis of retinal vein occlusion and (**b**) resolved cystoid macular oedema 2 months after the first dose of the intravitreal Ozurdex® implant

**Fig. 3:**
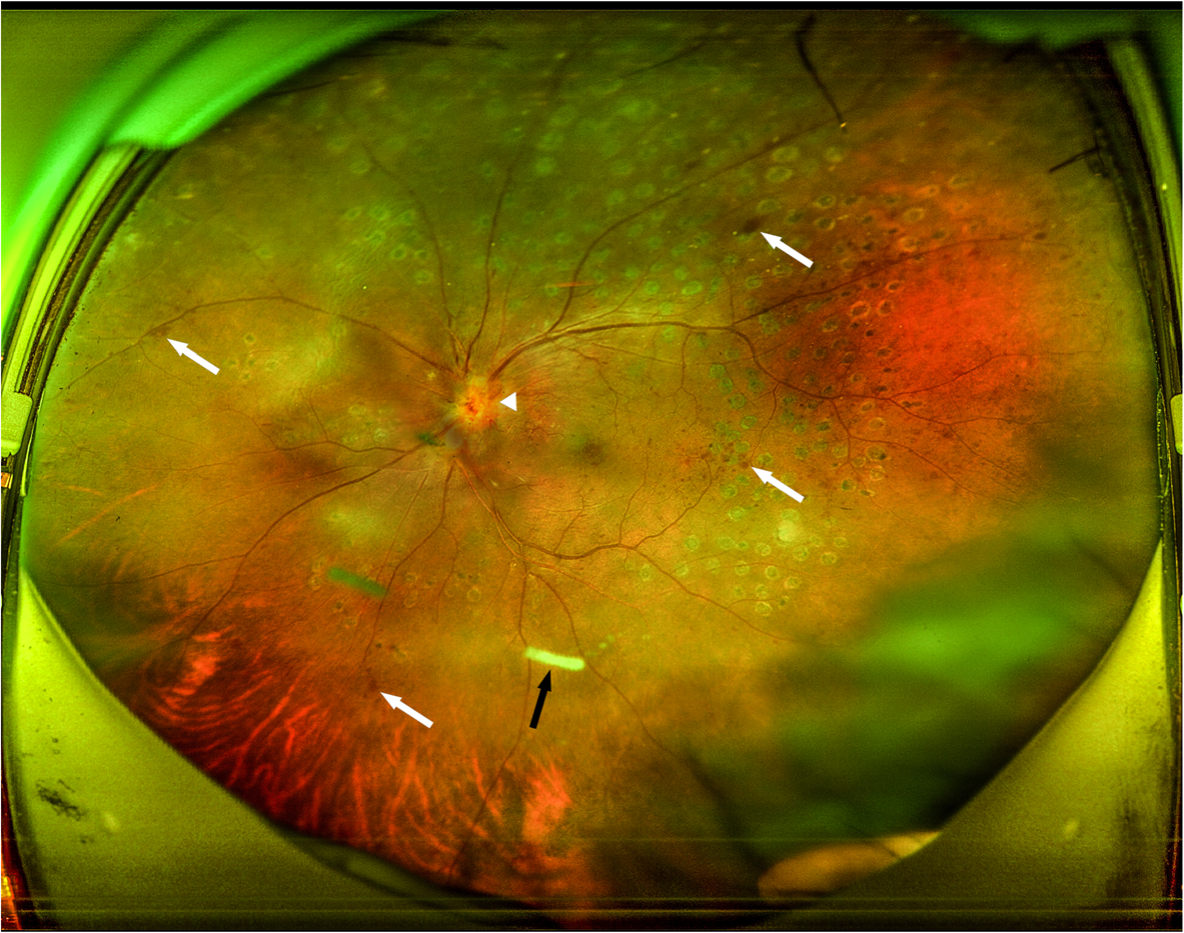
Ultra-widefield fundus photography of the left eye 1 week before diagnosis with acute retinal necrosis showing scattered retinal haemorrhages (white arrows), optic disc oedema (white arrow head), laser photocoagulation of the retina, and the intravitreal dexamethasone implant (Ozurdex®) (black arrow) in the inferior vitreous

A 45-year-old immunocompetent patient presented with photophobia, redness, floaters, and rapidly decreased vision in her left eye. She was diagnosed with HSV encephalitis more than 5 years ago via computed tomography scanning and magnetic resonance imaging of the brain, an electroencephalogram, positive serum IgG for HSV, and detection of HSV virus in the cerebrospinal fluid by polymerase chain reaction, she was cured at that time and stopped taking the relevant medicines. She received two injections of intravitreal Ozurdex® implant for macular edema from a central retinal vein occlusion (CRVO) three and 6 months prior to presentation (Figs 1, 2 and 3). Panretinal photocoagulation was performed 4 months prior to presentation. Her vision was 0.2 at the time of last Ozurdex® injection.

**Fig. 4:**
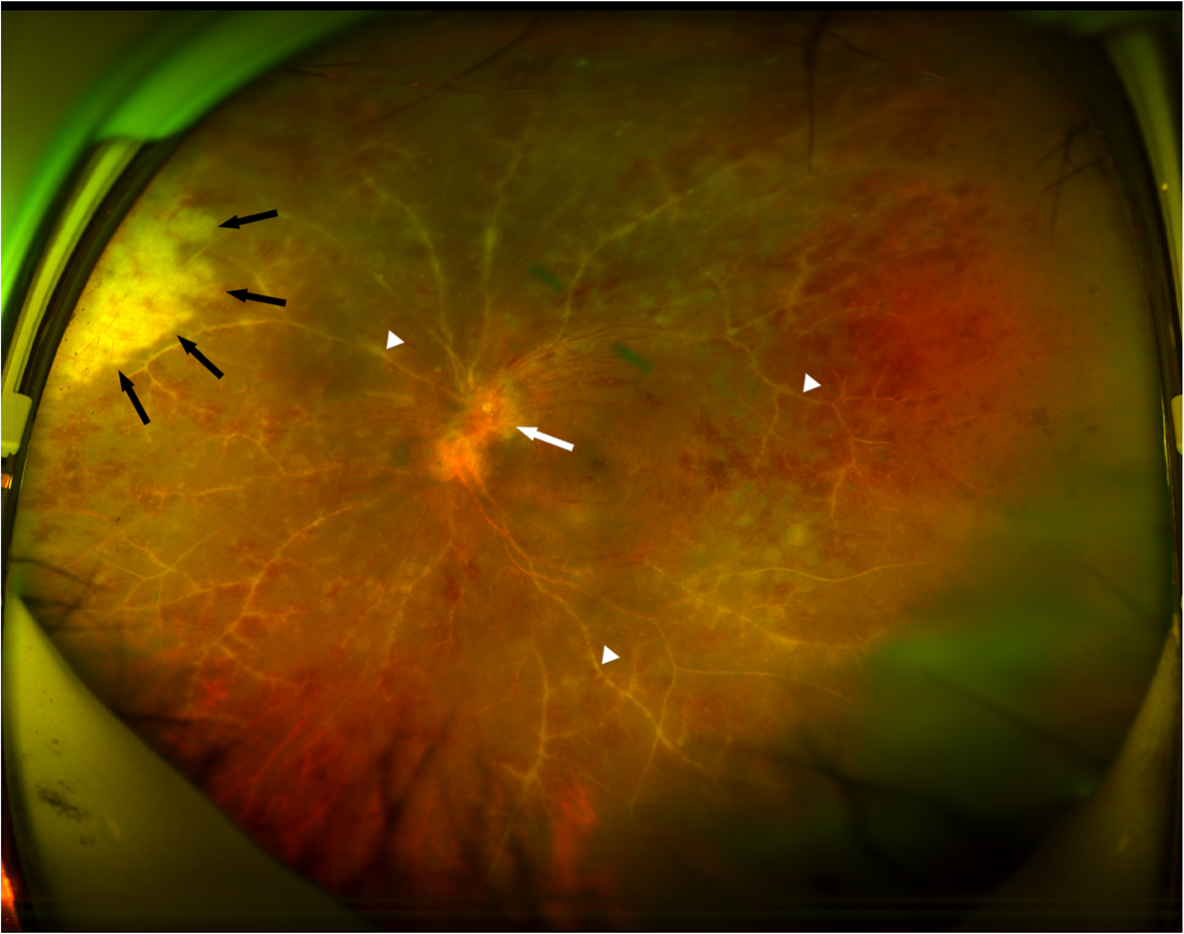
Ultra-widefield fundus photography of the left eye when diagnosed with acute retinal necrosis showing macular striation, optic disc oedema (white arrow), occlusion vasculitis (white arrow heads), retinal whitening at nasal periphery (black arrows)

**Fig. 5:**
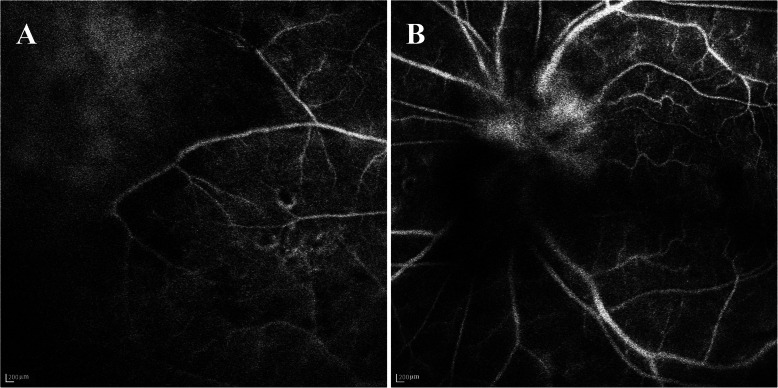
Fluorescein angiography images showing (**a**) retinal ischemia in the periphery and (**b**) optic disc leakage

Upon examination, her Snellen best-corrected visual acuity (BCVA) was 0.1 in the left eye. 2^+^ anterior chamber cells, granulomatous keratic precipitates, 3^+^ cells in the vitreous, optic disc oedema, occlusive retinal vasculitis, scattered retinal haemorrhages, and peripheral retinal whitening involving one quadrant were revealed (Fig. 4). The implant was observed in the inferior vitreous. Fluorescein angiography showed staining of the optic disc and vessels as well as the retinal nonperfusion zones (Fig. 5). Aqueous humour and vitreous samples were taken and analysed using quantitative polymerase chain reaction (PCR). The intravenous acyclovir treatment was initiated for a suspected herpes simplex or varicella-zoster-related ARN. The herpes simplex virus, but no varicells zoster virus, cytomegalovirus, or Epstein-Barr virus DNA, was detected in both the aqueous humour and vitreous samples via PCR. Additionally, the serology investigation was HSV-IgG positive and IgM negative. Systemic tests for syphilis, AIDS, and hepatitis were also negative.

**Fig. 6:**
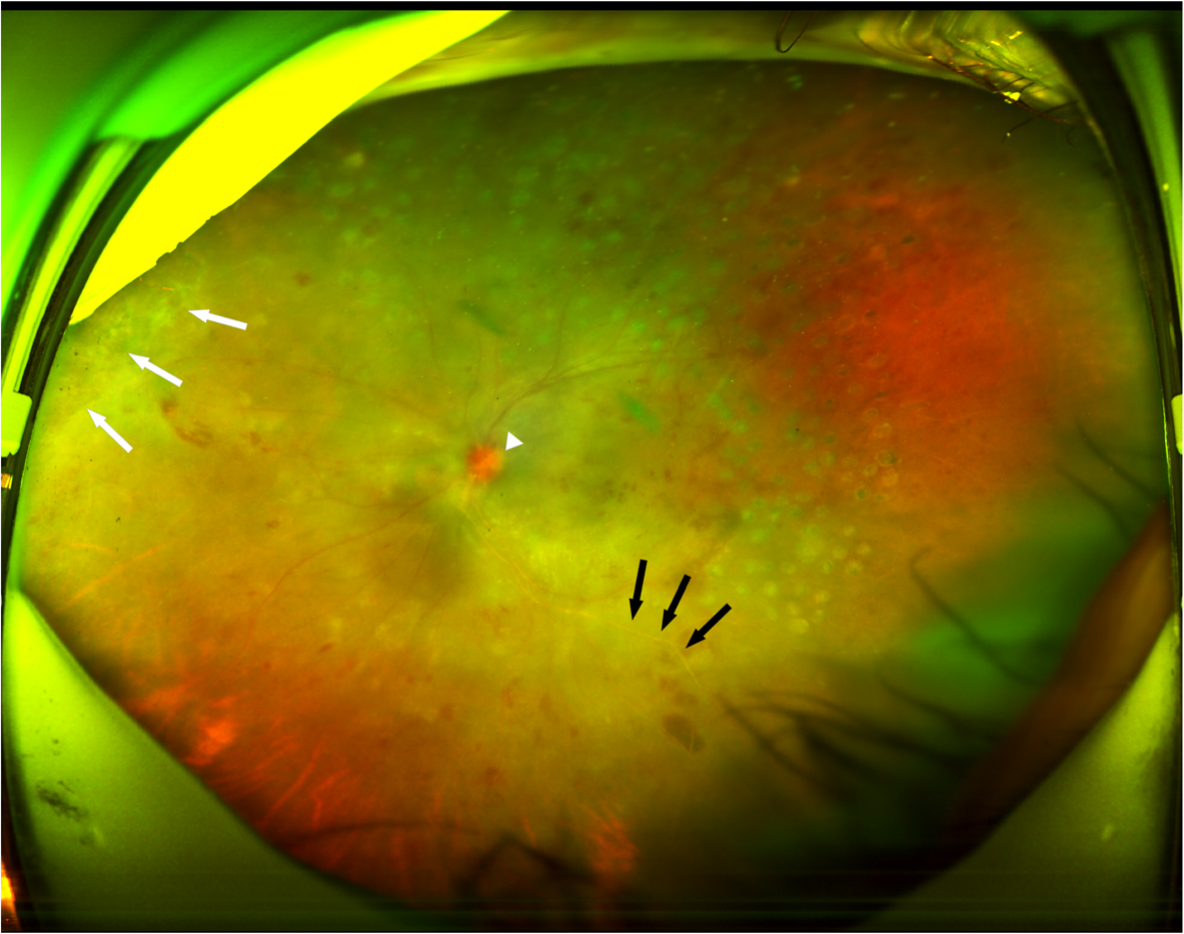
Ultra-widefield fundus photography of the left eye 4 months following presentation, demonstrating mild but reduced optic disc oedema (white arrow head), healing of the ARN area shown in Fig. 4 (white arrows), and new retinitis in the inferotemporal quadrant (black arrows)

The patient received 1% topical prednisolone acetate drops and 1% cyclopentolate drops. In addition, she received 500 mg intravenous acyclovir three times a day for 1 week, followed by 1000 mg oral valacyclovir three times a day until disease quiescence was achieved. After 4 months of treatment, granular hyperpigmentation was revealed in the affected retina (Fig. 6), and retinal detachment had not occurred. Additional pan-retinal photocoagulation was subsequently performed for the retinal nonperfusion zone. Prior to ARN, the patient’s Snellen BCVA was 0.3 in her left eye, and, after treatment with ARN for 4 months, her Snellen BCVA remained at 0.3. After that, the patient continued to take valacyclovir 1000 mg tid orally.

## Discussion and conclusions

ME is one of the major complications resulting in visual impairments in CRVO. One of the main goals in the clinical management of CRVO is to reduce ME and improve the patient’s vision [2]. Ozurdex® intravitreal injection, which is widely used in ME treatment, is the most direct method for delivering corticosteroids into the posterior segment. However, in the present report, intravitreal injected twice for recurrent ME resulted in ARN occurrence and worsening vision. Viral retinitis may develop following intraocular or periocular corticosteroid administration and has been reported in previous studies. The present case developed ARN about 3 months after her last intravitreal injection. The amount of time from corticosteroid (Ozurdex®) dosing to the onset of viral retinitis was similar to that which Takakura et al. [12] reported: 4.2 months (median 3.8; range 0.25–13.0). However, the causal role of intraocular corticosteroids in the development of viral retinitis is not fully understood, but most likely is the result of steroid induced immunesuppression. In addition, Ozurdex® has a long action timeframe of up to 4–6 months, which is different from the corticosteroids mentioned in previous studies [12].

The effective and prompt treatment of ARN patients is very important in terms of visual outcomes [15]. Historically, the diagnosis of ARN, which is a prerequisite for treatment, is made through clinical examination. According to aetiology and epidemiology reports, patients that present with ARN due to the VZV are often older, while those with HSV are much younger. Besides, HSV-induced retinitis typically affects younger patients who have a history of herpetic encephalitis [15].. Therefore, after initial clinical evaluation, our case with previous history of encephalitis was immediately treated with intravenous acyclovir. Then, the etiologic confirmation of HSV DNA was identified by analysing anterior chamber paracentesis and vitreous tap samples via PCR. One week after the first acyclovir injection, the patient’s Snellen BCVA of her ARN eye had recovered to 0.3, its pre-HSV infection value. Some previous reports have shown that the visual outcomes of ARN are poor [12, 16]. An ARN study with 15 patients indicated that one third of the eyes with BCVA could be deemed as legally blind, while 13% had improved, 63% remained stable, and 23% experienced visual deterioration [16]. Studies have also shown that the prognosis of visual acuity in patients with ARN might be related to the degree of initial visual acuity decline, etiological type, and neuropathy [17–19]. In our case, the initial present visual acuity only decreased to 0.1, the epidemiology was HSV infection, and only one retina quadrant was involved, so the visual prognosis of this patient was good. At the same time, the improvement of BCVA, in our case, indicated that the effective and prompt treatment of ARN patients after Ozurdex® implantation can prevent the disease from further deterioration, which is also very important in terms of visual outcomes.

However, the benefit of early laser retinopexy in lowering the risk of retinal detachment is controversial, with some authors suggesting that early laser retinopexy may lower the risk of retinal detachment [15, 16, 20]. Retinal necrosis causing retinal breaks is the main reasons leading to retinal detachments in affected eyes [19]. In previous reports, an aggressive initial involvement of more than two retinal quadrants was associated with more severe complications, such as retinal detachment [20, 21]. Therefore, the retinal photocoagulation before the occurrence of ARN and the subsequent additional photocoagulation could have prevented retinal detachment in this patient.

As is well-known, systemic and/or topical immunosuppression is an important risk factor for ARN occurrence. Intravitreal injections of triamcinolone, which might induce topical immunosuppression, have been described to be. associated with ARN. However, the effect of removing (or not removing) the implant on ARN treatment after the development of retinitis with topical immunosuppression is not fully understood. Takakura et al. [12] reported a successful treatment without removing fluocinolone acetonide (Retisert) implants after the development of retinitis. Considering that inflammatory factors play an important role in tissue destruction and that it can be controlled by the intravitreal injection of dexamethasone combined with antiviral treatment, some reports have used the intravitreal injection of dexamethasone in the management of ARN to promote recovery [22, 23] Our patient’s retinitis resolved after antiviral treatment without removing the implant, and visual acuity in the affected eye upon her last visit was equal to that before the onset of ARN. This might indicate that, not removing intravitreal corticosteroid implants, such as Ozurdex®, has no effect on effectively controlling retinitis via antiviral agents, on the contrary, it also helps inhibit the destruction of inflammatory factors.

The visual outcomes of ARN are usually poor, owing to severe ocular complications, such as retinal detachment, epiretinal membrane, and macular involvement by ischemic vasculopathy. Takakura et al. [12] reported that 14 out of 24 (58.4%) ARN cases have a BCVA worse than or equal to 0.1, and only 20.8% cases have a BCVA better than or equal to 0.5 upon the last visit. Although the visual outcome of ARN is related to several factors, including the occurrence of ocular complication and HSV or VZV causative agents, Miserocchi et al. [16] found that the number of retinal quadrants involved, vitreous haze had no influence on the rate of patients worsening or improving visual outcomes. In our case, BCVA improved to pre-ARN levels after 4 months of treatment. Although our case featured severe vitreous haze, the improving visual results might have not only benefited from prompt antiviral treatment but also from the lack of the macular complications and no retinal detachment.

Several studies have reported that HSV-ARN is associated with previous herpetic encephalitis or meningitis. Ganatra et al. [24] found that HSV-1-ARN is more frequently associated with a previous or concomitant history of encephalitis, while HSV-2-ARN is likely to be associated with a. previous or concomitant history of meningitis. Our case is in agreement with the literature regarding the association between encephalitis and HSV-ARN.

In summary, although intravitreal triamcinolone being associated with viral retinitis has been widely accepted, there are few reports on viral retinitis illuminating the unrecognized risk of Ozurdex® administration. Thus, the careful evaluation of factors such as history of encephalitis or meningitis and systemical or topical immunocompromization should be made before administering the intravitreal Ozurdex® implant.

## Data Availability

The datasets used and analysed during the current study are available from the corresponding author upon reasonable request.

## Abbreviations

ME: Macular oedema
RVO: Retinal vein occlusion
CRVO: Central retinal vein occlusion
ARN: Acute retinal necrosis
VZV: Varicella zoster virus
HSV: Herpes simplex virus
CMV: Cytomegalovirus
EBV: Epstein-Barr virus
BCVA: Best-corrected visual acuity
PCR: Polymerase chain reaction
